# Alternative approaches to tuberculosis treatment evaluation: the role of pragmatic trials

**DOI:** 10.1186/1745-6215-12-S1-A96

**Published:** 2011-12-13

**Authors:** Daniel J Bratton, Andrew J Nunn

**Affiliations:** 1 MRC Clinical Trials Unit, 125 Kingsway, London, WC2B 6NH, UK

## 

Clinical trials are sometimes classified as following either an explanatory or pragmatic design. Explanatory trials seek to investigate the efficacy of new treatments by imposing strict limitations on the design of the trial, including, for example, recruiting only those patients who are most likely to respond, considering only the most adherent patients in analyses or standardising trial treatment. In contrast, the objective of pragmatic trials is to assess whether treatments work in conditions more appropriate to routine practice and are therefore often much less restrictive in their design. This dichotomy is, however, over simplistic; there is a continuum which exists due to there being many areas of trial design that can vary between the extremities of the explanatory and pragmatic approaches.

The PRECIS (Pragmatic-Explanatory Continuum Indicator Summary) wheel is a tool that proposes a way of enabling researchers to assess the extent to which the trials they are designing could be considered explanatory or pragmatic. It is based on ten aspects of trial design, including inclusion criteria, flexibility of delivery of the intervention and intensity of follow-up. We consider how the PRECIS tool can be applied in the design stage of trials of tuberculosis treatment, to help teams design trials in line with their purpose. In view of the well recognised delay in getting results from well conducted clinical trials into practice, we would suggest that if more pragmatic trials are conducted, physicians would better understand the implications of the results for their own practice and be more ready to adopt new treatments [1].

## References

[B1] Bratton DJ Nunn AJ Alternative approaches to tuberculosis treatment evaluation: the role of pragmatic trials Int J Tuberc Lung Dis 2011 15 440 446 10.5588/ijtld.10.0732 21396200

